# Supplementary material for: The impact of different antimicrobial exposures on the gut microbiome in the ARMORD observational study
Source: eLife. 2025 Dec 10;13:RP97751. doi: 10.7554/eLife.97751 (PMC12695023; doi:10.7554/eLife.97751)
Supplement: Supplementary file 1. [file elife-97751-supp1.docx]

# Case Report Form

**Antibiotic Resistance in the Microbiome OxfoRD (ARMORD) Study – Study Questionnaire for investigator completion**

version 3.0 (25^th^ November 2015) Author: N Fawcett

THIS IS A RESEARCH STUDY OFFICIAL DOCUMENT AND IS HIGHLY CONFIDENTIAL. IF YOU ARE NOT A MEMBER OF THE ARMORd RESEARCH TEAM AND HAVE OBTAINED THIS DOCUMENT IN ERROR, PLEASE INFORM THE RESEARCH TEAM IMMEDIATELY ON: [armord@ndm.ox.ac.uk](mailto:armord@ndm.ox.ac.uk) /01865 222194, or hand it in to the Microbiology Department on Level 7 of the John Radcliffe Hospital (for attention of Prof Crook Group). We will pick it up and review our handling of this document.

**Please confirm with the participant this statement: “Whilst this information is of great help to us in understanding antibiotic resistance and the microbiome, you are free to choose which questions to answer, and if you require clarification or prefer not to answer, let us know”.**

**Study Number: ___________________ Date of Questionnaire: _____________**

**Researcher: ______________________**

1. **Tell me about any antibiotics you may have received in the last year:**

Antibiotics in past month? Y / N

Antibiotics in the past year? Y / N

If yes to either then use additional data sheet

1. **Tell me anything you can recall about your antibiotic use throughout your life. For example - we would like to find out if you have a condition like asthma and may have taken many courses of antibiotics every year, or if you are someone who virtually never takes antibiotics.**

Antibiotic use: More than 10 courses in the last 5 years [ ]

Antibiotic use: 1 – 10 courses in the last 5 years [ ]

Antibiotic use: No antibiotics in the last 5 years [ ]

Notes:

1. **Have you had any infections (such as urinary tract infections, pneumonia or skin infections) in the last 3 years?**

Had an infection, think it was resistant [ ]

Had an infection, think it was sensitive [ ]

Had an infection, but don’t know the resistance information [ ]

No infection [ ]

Details (what do you know about it?)**:**

1. **Tell me about any travel in the last three years:**

No Travel outside UK [ ]

Travel outside UK [ ] (use additional data sheet)

1. **What best describes your diet?**

No dietary restrictions [ ]

Vegetarian (eats eggs & milk) [ ]

Vegan (no eggs or milk products) [ ]

1. **How often do you touch or handle the following food products?**

|  | Never | Occasionally/once a month or less | Once a week to once a month | Multiple times a week |
| --- | --- | --- | --- | --- |
| Raw chicken meat |  |  |  |  |
| Other raw meat: |  |  |  |  |
| Raw shellfish: (including prawns) |  |  |  |  |

1. **Do you work in the food industry?**

No [ ] Animal farm [ ] Plant farm [ ] Abattoir [ ]

1. **Do you work in the healthcare profession?**

No [ ]

Hospital, direct physical contact with patients [ ]

Hospital, no direct contact with patients [ ]

Hospital including patient bodily hygiene and contact with waste matter [ ]

GP surgery/outpatient clinic* only, direct contact with patients [ ]

GP surgery/clinic*, no direct contact with patients [ ]

*a healthcare site which doesn’t have patients to stay overnight for care

1. **How often have you been to hospital in the last year?**

No contact [ ]

As a patient:

Contact, only outpatients/day case [ ]

Contact, inpatient [ ]

Last date of contact:

| How much? | 1 day [ ] | 2-14 days [ ] | >14 days [ ] |
| --- | --- | --- | --- |

Details:

Do you visit others in hospital regularly?

Notes (place/duration):

1. **Have you been admitted to hospital overnight or more in the last 5 years?**

No [ ] Yes [ ] (use additional data sheet)

1. **How often have you been in contact with residential/nursing care in the last year?**

As a resident:

Never [ ] Previous contact, not current [ ] Current [ ]

Notes (last contact, type of care, total approx. duration):

As a visitor:

| How often as a visitor? | Never | Occasionally/once a month or less | Once a week to once a month | Multiple times a week | Approx. date of last contact |
| --- | --- | --- | --- | --- | --- |
| Nursing Care |  |  |  |  |  |
| Residential care |  |  |  |  |  |

1. **How often do you provide childcare for babies or children <2 years?**

|  | Never | Occasionally/once a month or less | Once a week to once a month | Multiple times a week | Approx. date of last contact |
| --- | --- | --- | --- | --- | --- |
| Any care |  |  |  |  |  |
| Care, including personal hygiene for child (body washing and/or handling of nappies) |  |  |  |  |  |

1. **Do you have any pets in your household?**

Cat [ ]

Dog [ ]

Other(s) [ ] (please specify):

None [ ]

I clean up their poo Y / N

1. **Have you had any episodes of diarrhoea in the last 2 months?**

No [ ]

1+ episode of diarrhoea [ ]

Loose stools on daily basis [ ]

Notes (cause if known, timing):

1. **Have you been treated with chemotherapy, radiotherapy or medication which affects the immune system* in the last 5 years?**

No [ ]

Yes [ ]

Don’t know [ ]

Notes (What was it for? How long did you take it for? How did you get given it? e.g. tablets/injections):

* You may have received this if you had an autoimmune disease (overactive immune system) This includes steroids, methotrexate, hydroxychloroquine, sulfasalazine, leflunomide, and injections of anti-TNFa/IL-2R treatment for conditions like rheumatoid arthritis.

1. **Have you taken any of the following in the last month?**

| Medication *(if in doubt , you can discuss at the visit)* | Never | Yes, regularly | Occasionally | Don’t know |
| --- | --- | --- | --- | --- |
| Proton-pump inhibitors (e.g. Omeprazole, lansoprazole) |  |  |  |  |
| Non-steroidal anti-inflammatory drugs (e.g. ibuprofen, aspirin, diclofenac, naproxen) |  |  |  |  |
| Vitamin, herbal or dietary supplements (including fibre) |  |  |  |  |

Notes (What was it called? What was it for? How long did you take it for? How did you get given it? e.g. tablets/injections):

1. **How often do you eat the following foods?**

(if you are tired or short of time, we can leave it or go through this at another visit)

| Type of food (approx. serving size) | Never | Less than once a week | Once a week | 2-4 times a week | 5-6 times a week | Once or more daily | If more than once daily – how many times? |
| --- | --- | --- | --- | --- | --- | --- | --- |
| High fibre food – brown bread, brown rice, high fibre cereal (one handful/piece of bread) |  |  |  |  |  |  |  |
| Other cereals, breads potatoes, rice, pasta (one handful/piece of bread) |  |  |  |  |  |  |  |
| Fresh fruit (one apple size) |  |  |  |  |  |  |  |
| Fresh vegetables (one apple size) |  |  |  |  |  |  |  |
| Milk, cheese and yoghurt products (one glass/ small yoghurt pot) |  |  |  |  |  |  |  |
| Meat, fish and poultry (one handful) |  |  |  |  |  |  |  |
| Non-animal meat alternatives (Quorn, soy mince) (one handful) |  |  |  |  |  |  |  |
| Eggs |  |  |  |  |  |  |  |
| Oil and butter (one tablespoon) |  |  |  |  |  |  |  |
| Chocolate, cake and sweets (one chocolate bar/handful) |  |  |  |  |  |  |  |
| Ready meals/pre-packaged meals |  |  |  |  |  |  |  |

1. **Have you ever smoked as much as one cigarette a day for as long as a year?**

No (non-smoker) [ ]

Yes:

I have smoked this amount previously but have not smoked a cigarette in the last year [ ]

I have smoked less than one cigarette a day in the last year [ ]

I currently smoke one cigarette or more, on average, daily [ ]

Notes:

1. **How many units a week do you drink of alcohol (on average in the last year)?**

(pint of beer = 2 units, small glass of wine = 1 unit, large glass of wine = 2 units, shot of spirits = 1 unit)

Units/week:

Never drink alcohol [ ]

1. **Do you have anything else of relevance you think we should know?**

**ARMORD additional data sheet for investigator (enter approximate values if unknown)**

| **Antimicrobial**  **(non-OUH in past year, U = unknown)** | **Route** | **Start date** | **Stop date or  duration (d/w/m)** | **Indication**  **(blank if unknown)** |
| --- | --- | --- | --- | --- |
|  |  |  |  |  |
|  |  |  |  |  |
|  |  |  |  |  |
|  |  |  |  |  |
|  |  |  |  |  |
|  |  |  |  |  |
|  |  |  |  |  |
|  |  |  |  |  |
|  |  |  |  |  |
|  |  |  |  |  |
| **Hospital**  **(admissions outside OUH in past 5 years)** | | **Admission** | **Discharge or  duration (d/w/m)** | **Reason**  **(blank if unknown)** |
|  | |  |  |  |
|  | |  |  |  |
|  | |  |  |  |
|  | |  |  |  |
|  | |  |  |  |
|  | |  |  |  |
|  | |  |  |  |
| **Country**  **(past 3 years, record only last visit to each country)** | | | **Start** | **End or**  **duration (d/w/m)** |
|  | | |  |  |
|  | | |  |  |
|  | | |  |  |
|  | | |  |  |
|  | | |  |  |
|  | | |  |  |
|  | | |  |  |
|  | | |  |  |
